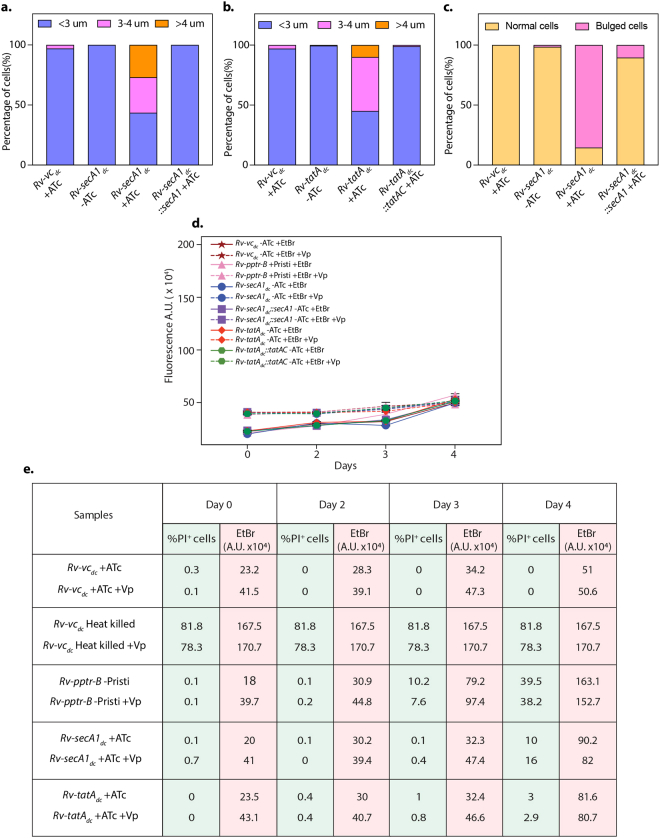# Sec and Tat Mediated Secretion Safeguards *Mycobacterium tuberculosis* Membrane Homeostasis

**DOI:** 10.1016/j.mcpro.2026.101555

**Published:** 2026-03-17

**Authors:** Priyadarshini Sanyal, Jagadeeshwari Uppada, Shashank Sinha, Yashasvi Bhat, Sidra Khan, Shri Vishalini Rajaram, Evanjalee Albert Arokiyaraj, Gagan Deep Jhingan, Nisheeth Agarwal, Areejit Samal, Vinay Kumar Nandicoori

**Affiliations:** 1 CSIR-Centre for Cellular and Molecular Biology (CSIR-CCMB), Hyderabad, Telangana, India; 2 Academy of Scientific and Innovative Research (AcSIR), Ghaziabad, Uttar Pradesh, India; 3 The Institute of Mathematical Sciences (IMSc), Chennai, Tamil Nadu, India; 4 BRIC-National Institute of Immunology (BRIC-NII), New Delhi, India; 5 Center for Biotechnology, Anna University, Chennai, Tamil Nadu, India; 6 BRIC-Translational Health Science and Technology Institute (BRIC-THSTI), Faridabad, Haryana, India; 7 Homi Bhabha National Institute (HBNI), Mumbai, Maharashtra, India

**Keywords:** CRISPRi knockdown, membrane permeability, membrane proteome, protein secretion, secretome, secretory components

## Abstract

Protein secretion is essential for the growth and virulence of *Mycobacterium tuberculosis*, yet the organization and function of its secretion pathways remain poorly understood. We reviewed the existing literature, combined it with systematic queries, and finalized annotations based on experimental data and computational predictions to compile a curated list of 92 secretory components and 198 reactions involved in Sec, twin-arginine translocation (Tat), and ESX pathways. Using CRISPRi, targeted depletion of SecA1 or TatAC impaired both *in vitro* growth and *ex vivo* survival. Label-free quantitative secretome analysis revealed decreased export of substrates dependent on SecA1 and TatAC, with enrichment of cytosolic proteins in culture filtrates, indicating increased membrane dysbiosis. Membrane proteomics showed elevated levels of proteins engaged in intermediary and lipid metabolism, while proteins associated with the cell wall and cell processes decreased, suggesting weakened membrane integrity. Loss of SecA1 or TatAC increased membrane permeability, with the effect being more pronounced in the case of TatAC, and caused structural abnormalities seen under electron microscopy. Overall, our integrated multi-omics and functional genetics studies demonstrate that the SecA1 and Tat pathways are essential for maintaining membrane homeostasis in *Mycobacterium tuberculosis*. These results suggest that essential secretory proteins may be promising targets for therapeutic intervention.

## Highlights

• Computational Secretion Systems map in Mtb: 92 components, 198 reactions across Mtb secretion and TF–omics overlay defines regulation of secretory gene networks.
• CRISPRi-mediated depletion of SecA1 or TatA significantly reduced bacterial survival *in vitro* and *ex vivo*, establishing their essential roles in Mtb physiology.
• Quantitative secretomics, membrane proteomics and phenotypic analyses, including ultrastructural defects and increased EtBr uptake, demonstrated disrupted membrane homeostasis upon SecA1 or TatAC depletion.

## Graphical Abstract

**Figure:**
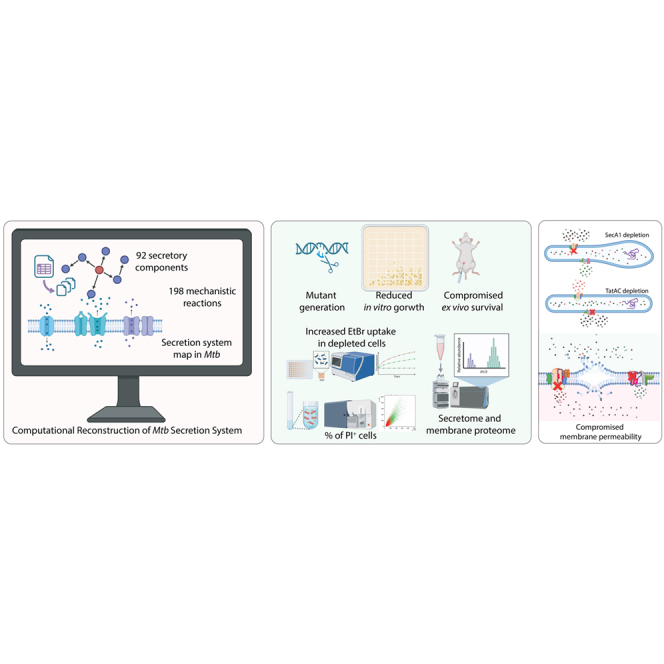

## In Brief

The organization and function of secretion pathways in *Mycobacterium tuberculosis* (Mtb) remain unclear. We reconstructed a computational secretory system map for Sec, Tat, and ESX pathways in Mtb containing 92 secretory components and 198 reactions. CRISPRi, depletion of SecA1 or TatAC impaired bacterial survival. Quantitative secretome and membrane proteome revealed enrichment of cytosolic proteins suggesting weakened membrane integrity of depleted strains. SecA1 or TatAC loss increased permeability and structural defects, indicating their essential roles in membrane homeostasis and therapeutic targeting potential.

Protein secretion is a ubiquitous mechanism for transporting macromolecules across membranes. It is found in all three domains of life: *Prokaryota, Archaea, and Eukaryota*. In Eukaryotes, protein secretion occurs through a general pathway called the “Sec pathway.” This pathway is present in the endoplasmic reticulum membrane and involves secretory vesicles carrying proteins to the Golgi apparatus (1). In prokaryotes, proteins with N-terminal signal peptides (SP) are mainly secreted through two pathways. These are the Sec-dependent pathway (2, 3) and the Sec-independent, twin-arginine translocation pathway (Tat) (4), which is also conserved in plants (5, 6, 7, 8).

Sec-dependent secretion is divided into two types: a) cotranslational translocation (Signal Recognition Particle (SRP)-mediated), which exports unfolded proteins while being translated by ribosomes, and b) post-translational translocation (SecA1 and SecA2-mediated), which exports unfolded proteins post-translation (9). The Sec secretion system comprises SecY, SecE, and SecG proteins (9, 10, 11), which form the SecYEG-SecDF-YajC-YidC holotranslocon, with the help of auxiliary proteins SecD, SecF, YajC, and YidC (12). SRP and SecA recognize nascent preproteins with an N-terminal Sec SP, directing translocation *via* GTP hydrolysis of FtsY in the SRP pathway and ATPase activity of SecA in the case of SecA-mediated transport (13). Type I and II signal peptidases; associated with the holotranslocon, act on the cleavage sites, facilitating the conversion to a mature protein (8, 14). The Tat (15) system in prokaryotes and the thylakoid membrane of plant chloroplasts (4) is involved in secreting a subset of folded proteins (15, 16) containing two invariant arginine residues (RR motif) (17). In *Escherichia coli*, five *tat* genes, *that is tatABCDE*, have been reported, among them *tatABCD* are present in an operon (18).

*Mycobacterium tuberculosis* (*Mtb*) secretes an array of proteins, including virulence factors, with the help of generalized and specialized secretion pathways. In *Mtb*, the canonical SecA has two paralogs, SecA1 and SecA2 (19, 20) both having ATPase activity; the non-canonical SecA2 differs from SecA1 in that it only secretes a small set of proteins, which are known to be involved in virulence (21, 22, 23, 24). The *Mtb* genome does not encode *tatE*, and *tatD* is not involved in Tat-mediated protein export (18). TatB and TatC form a Tat Complex-I in the inner membrane, and during translocation, binding of TatA forms Tat Complex-II, which forms an oligomeric pore across the membrane that assists in proton motive force mediated protein translocation (25). Specialized secretion systems like Type VII Secretion Systems (T7SS) transport small proteins with PE/PPE, WXG, or YXXXD/E motifs (26) and comprise five systems (ESX-1 to ESX-5) that are involved in virulence, pathogenesis, metal homeostasis, and host modulation (27). While sharing common structural and evolutionary features, ESX-1, ESX-3, and ESX-5 are well-studied and functionally linked, whereas ESX-2 and ESX-4, which contribute to DNA regulation (28) and heme acquisition (29), are less characterized.

There are several gaps in our understanding of this complex network of secretion systems in *Mtb*, which we aimed to address. We first considered what is known about: (i) the core and accessory components of different secretion systems and their organization, and (ii) the involvement and regulation of these components across multiple stages of protein transport, to establish the conceptual framework for our study. Building on this foundation, we asked experimentally: (iii) what is the role of the SecA1 and Tat secretion systems in cell growth and survival, and what are their substrates? (iv) what is the impact of depleting SecA1 and TatAC on *Mtb* membrane homeostasis? In this study, we systematically reviewed the published literature and integrated it with ChIP-Seq and transcriptomic datasets to create an *in silico* systems-level map of secretory pathways and uncovered the network’s regulatory structure. We generated conditional depletion strains of SecA1 and the Tat pathway to explore their biological roles. Proteome analysis of WT and depleted strains suggested that although secretion of target substrates is compromised, it leads to the unusual secretion of cytosolic proteins. Experiments indicate that depletion of these systems impacts membrane homeostasis, reflected in changes in morphology and permeability.

## Experimental Procedures

### Bacterial Strains and Plasmids Used for the Study

*Mtb H37Rv* (*Rv*), a pathogenic laboratory strain sensitive to all antibiotics, was used in this study. Conditional knockdown strains *Rv-secA1*_*dc*_*, Rv-tatA*_*dc*_*,* and complementation strains were generated by electroporating relevant constructs into *Rv*. The pTetIntdCas9 plasmid used in this study has dCas9 under a tetracycline (ATc) inducible promoter and an L5 integrative site, as described previously (30). Two guide RNAs were designed to achieve sufficient depletion upon the addition of ATc (500 ng/ml). *sec1144* and *sec1183* are the guide RNA sequences aimed at targeting *secA1*, and *tat1142* and *tat1181* are two guide RNA sequences for *tatA*. Two guide RNAs, each of 20 nucleotides complementary sequence for each target gene from 5′UTR and after the PAM sequence 5′NGG3′ were cloned into the pTetIntdCas9 vector pTet-dC-SecA1 and pTet-dC-TatA (Supplemental Fig. S5, *A* and *B*). Expression of dCas9 and target sequence-specific guide RNAs, both under a Tet-inducible promoter, halts RNAP progression; thus, the target gene is knocked down (Supplemental Fig. S5*C*). For complementation, the *secA1 (secA1*_*gmut*_*)* or *tatA* (*tatA*_*gmut*_) were mutated (Supplemental Fig. S5*D*) such that the guide RNA binding is abrogated. *secA1*_*gmut*_ and its native promoter were amplified separately, digested, and cloned into the pSNG-S vector containing giles attP and Integrase (unpublished) to generate pSNG-secA1_gmut_. *tatA*_*gmut*_*,* along with downstream *tatC* and their native promoter, were amplified separately (Supplemental Fig. S5*D*), digested, and cloned into the pSNG-S vector to generate pSNG-tatAC_gmut_. The *pknB* conditional gene expression strain under regulation of *pptr* promoter (Pristinamycin) *Rv-pptr-B* was used as a positive control (31).

### Antibody Generation, Lysate Preparation, and Western Blots

The antibodies (SecA1 and TatA) are polyclonal. 6x-His-tagged full-length proteins SecA1 and TatA were purified using Ni-NTA beads and injected into mice. Serum was collected and tested for antibodies using *Mtb* WT lysate at different dilutions. To evaluate the antibodies raised, 20 ng of purified TatA/SecA1 were resolved and probed with α-TatA (5000 diluted) and α-SecA1 (10,000 diluted). It is apparent from the full blots provided in Panel one and Panel three that the antibodies raised could effectively recognize 20 ng of proteins (Supplemental Fig. S5*F*). To evaluate their ability to detect endogenous SecA1 and TatA proteins, we have resolved 20 μg and 100 μg of Rv lysate, respectively. Membranes were probed with α-SecA1 antibody (1:5000 dilutions) and α-TatA antibody (1:1000 dilutions). While we detected a single band with α-SecA1 antibody, we observed a few contaminating bands when we probed with α-TatA antibody (Supplemental Fig. S5*F*). However, the most prominent band corresponded to TatA, and hence we used these antibodies for further work. *Rv-vc*_*dc*_*, Rv-secA1*_*dc*_*, and Rv-tatA*_*dc*_ strains were grown until A_600_ ∼0.8, and whole cell lysate (WCL) was prepared by resuspending the cell pellet in lysis buffer (1× PBSG, 1× PMSF, and PIC: Protease Inhibitor Cocktail). The cells were lysed using bead beating (8–10 cycles, 1 min ON and 2 min OFF). The cells were centrifuged at 158,71*g* for 10 min at 4 °C, and the supernatant was quantified using the BCA reagent (Thermo Fisher Scientific). The WCLs were resolved on 10% (for SecA1) or 15% (for TatA) SDS-PAGE gels, transferred to nitrocellulose membranes, and probed with α-SecA1 or α-TatA, or α-GroEL1 antibodies raised in the lab.

### RNA Isolation and qRT-PCR

The *Rv-vc*_*dc*_*, Rv-secA1*_*dc*_*, and Rv-tatA*_*dc*_ strains, with or without ATc, were grown to A_600_ ∼0.8, and cells equivalent to 10 O.D. were harvested after 3 days of ATc treatment. The cell pellets were resuspended in 1 ml of Trizol (Invitrogen), and cells were lysed by bead beating (3 rounds of 30 s ON and 2 min OFF). The cells were centrifuged at 158,71*g* for 10 min at 4 °C, and 300 μl of chloroform was added to the supernatants. The samples were vortexed and centrifuged, and the aqueous layer containing RNA was precipitated. The pellet was resuspended in RNase-free water, treated with DNase I (Invitrogen), and purified using a Qiagen column. 1 μg of RNA was reverse transcribed using the iScript cDNA synthesis kit (Bio-Rad). Quantitative real-time PCR was performed with iTaq Universal SYBR Green Supermix (Bio-Rad) on the QuantStudio three system (Applied Biosystems). Data were normalized to 16S rRNA expression levels. The ΔΔCt algorithm was used to evaluate the relative fold change in gene expression.

### Bacterial Survival *invitro* and upon *exvivo* Peritoneal Macrophage Infection

The strains were inoculated at A_600_ ∼0.1 in the presence and absence of ATc. The colony forming units were enumerated on day 0, 2 and 4. Colonies were calculated and plotted using GraphPad Prism. For the *ex vivo* infections, peritoneal macrophages were used. Briefly, thioglycolate was injected into the mouse peritoneum, and macrophages were isolated 4 days post-injection. All the procedures involving live animal were conducted following the regulations and ethical guidelines approved by Institutional Animal Ethics Committee at CSIR-CCMB animal house facility (Institutional Animal Ethics Committee **-**25/2023). 3 × 10^5^ cells were seeded per well, and infection was performed at a 1:5 (macrophage:bacterial cells) MOI. Colony forming units were enumerated at 4 and 72 h post-infection with and without ATc. Colonies were counted, and a graph was plotted using GraphPad Prism.

### Computational Reconstruction of Secretory Systems in *Mtb*

**Figure S1:**
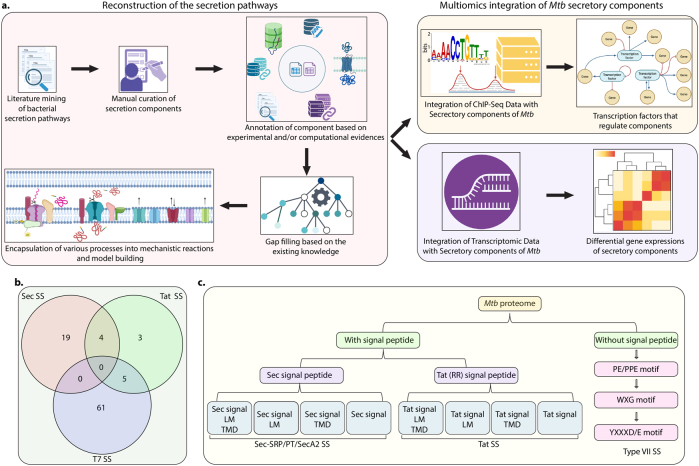


Inspired by the work by Feizi *et al.* on genome-scale reconstruction of the protein secretion system in yeast *Saccharomyces cerevisiae*, we created a manually curated reconstruction of the secretion systems in *Mtb* (32). The systematic manual curation of existing literature on bacterial secretion systems, supported by both experimental and/or computational evidence, was performed as depicted in the workflow (32, 33) (Supplemental Fig. S1*A*). Based on the compiled evidence, components of secretion pathways, namely, Sec-dependent (SRP-dependent, SecA1-dependent, and SecA2-dependent), Sec-independent (Tat), and T7SS, were collated and annotated (Supplemental Tables S1 and S2).

**Fig. 1:**
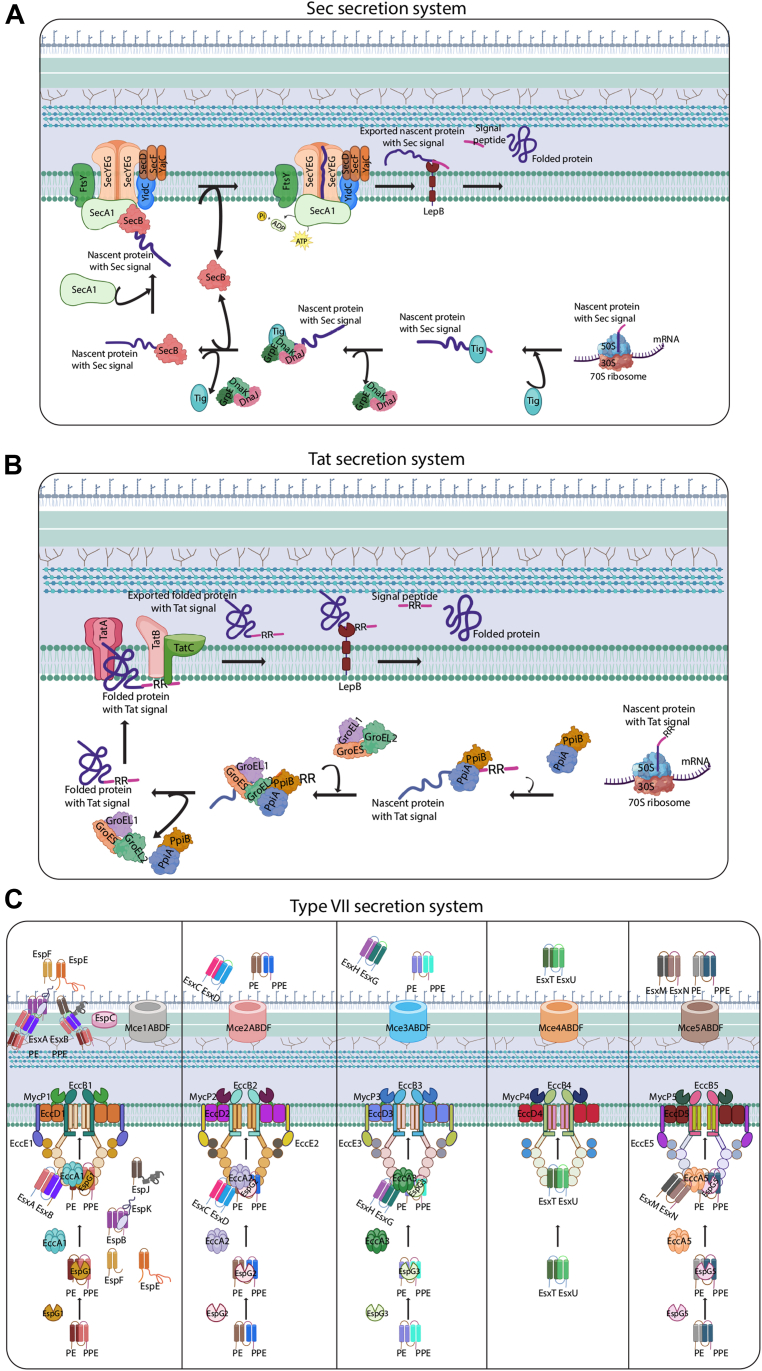
**Schematic representation of secretion systems in *Mt*b.***A*, pictorial model depicting the steps involved in the secretion of a protein containing an N-terminal Sec-specific signal peptide using the SecA1-dependent Sec pathway. *B*, steps involved in the secretion of a protein containing a Tat signal peptide (twin arginine -RR- motifs) using the Tat pathway. *C*, overview of protein secretion using specialized secretion systems (ESX1-ESX5) in *Mtb*. The steps shown were based on mechanistic reactions derived from the experimental evidence provided in Supplemental Table S2. Created in BioRender. Nandicoori, V. (2026) https://BioRender.com/au2hqr4.

**Figure S2:**
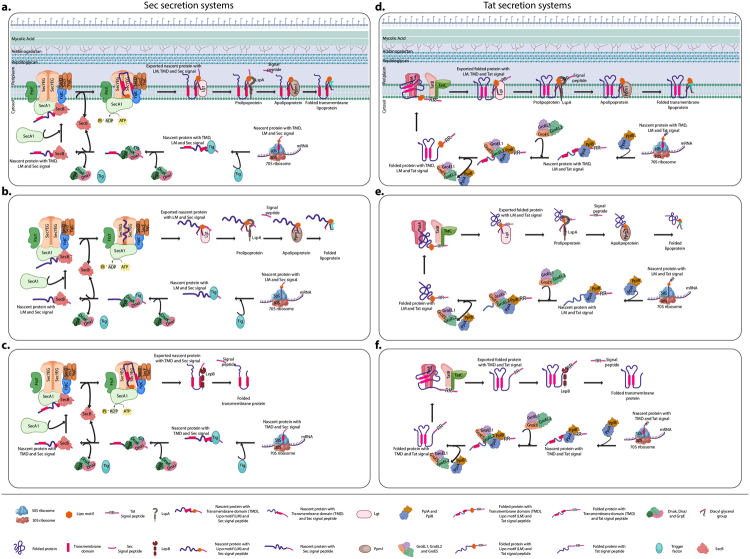


**Figure S3:**
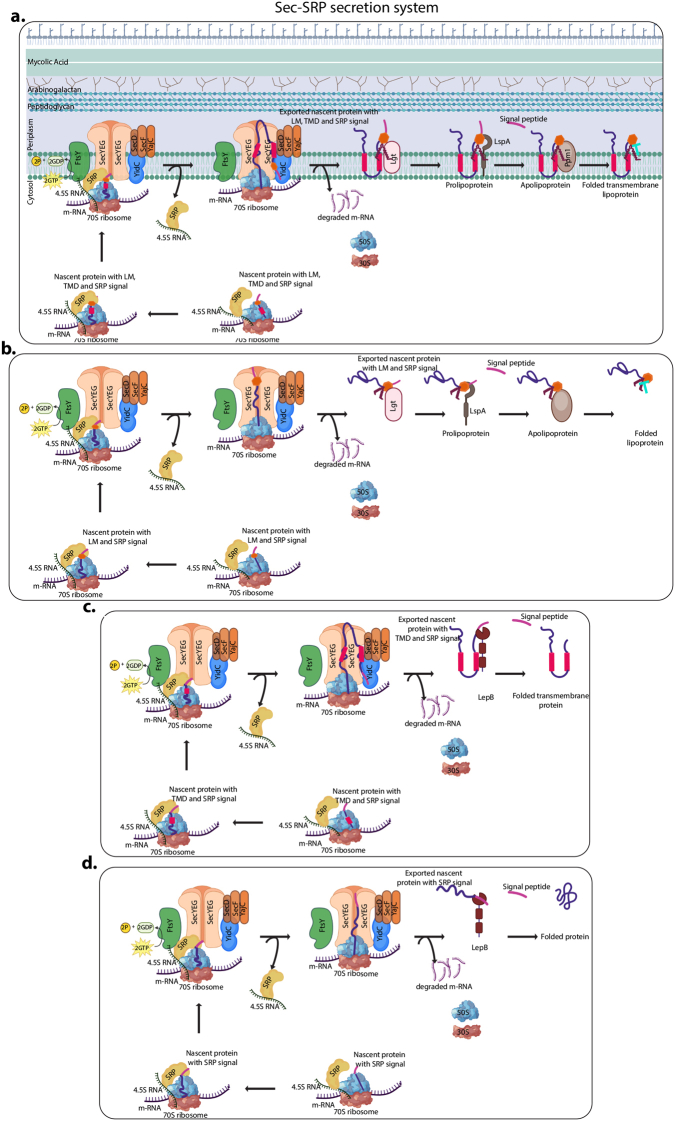


Further, the protein signatures relevant for secretion and localization, such as presence or absence of SP sequences, lipo motif (LM), transmembrane domain, etc., were assigned using the computational tools SignalP v6.0 (34), Phobius v10.1 (https://phobius.sbc.su.se/), DeepTMHMM v1.0 (https://services.healthtech.dtu.dk/services/DeepTMHMM-1.0/), and PcnsHub (35), facilitating the identification and classification of secreted proteins (Supplemental Fig. S1*C*). Detailed mechanistic information from the existing literature was used to further organize the secretion process into a series of pseudo-chemical reactions, similar to reactions formulated by Feizi *et al.* for the classical secretion pathway in yeast (32). A pictorial representation of these reactions was created using Biorender (Fig. 1, Supplemental Figs. S2, and S3).

**Figure S4:**
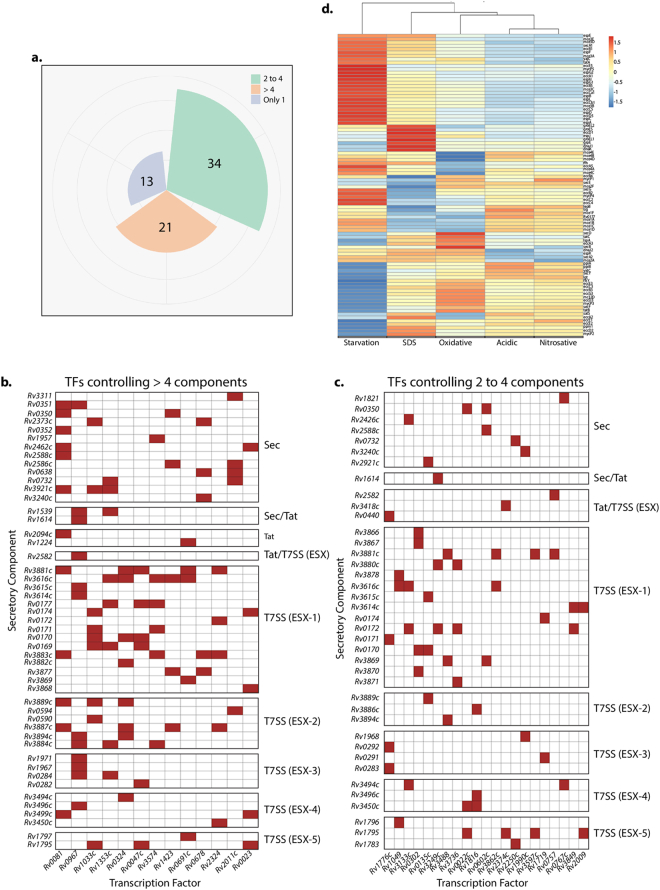


To explore the transcriptional regulation of secretory components, publicly available ChIP-Seq data (36) for *Mtb* transcription factors (TFs) was used to identify TFs directly controlling the different *Mtb* secretory components (Supplemental Fig. S4 and Supplemental Table S3). A visualization of TFs controlling the *Mtb* secretory components was generated using the R package ggplot2 (https://ggplot2.tidyverse.org/). Gene-level expression changes in secretory components under different stress conditions were obtained from a transcriptomic study by *Khan et al.* (37). Differential expression was visualized using a heatmap generated with the R package pheatmap (https://cran.r-project.org/web/packages/pheatmap/index.html).

### Culture Filtrate Protein Preparation

To isolate the culture filtrate (CF) proteins from *Mtb*, *Rv-vc*_*dc*_*, Rv-secA1*_*dc*_*,* and *Rv-tatA*_*dc*_*, cultures* were grown to early log phase, with or without ATc (500 ng/ml), in Sauton’s media in quadruplets (38). Briefly, the cells were harvested and centrifuged at 1503*g* for 10 min at 4 °C. The supernatants were collected into a fresh 50 ml Falcon tube, and the pellets were kept aside for preparation of WCL (Whole Cell Lysate). The supernatants were centrifuged again under the same conditions, and the procedure was repeated twice. The supernatants were collected and passed through a 0.2 μm syringe filter after the last centrifugation. The filtered supernatants (CF proteins) were concentrated using 3 kDa Amicon centricons (∼150 ml CF to 1 ml) and stored at −80 °C (Supplemental Fig. S6*A*).

### Secretome Sample Preparation for Mass Spectrometry

For mass spectrometry of CF proteins, 100 μg were taken into fresh low protein-binding MCTs (microcentrifuge tubes), and 6× volume of ice-cold acetone was added for overnight precipitation at −20 °C. The samples were centrifuged at 158,71*g* for 10 min at 4 °C, the supernatant was discarded, and the pellets were dried in a speed vacuum. 100 μl resuspension buffer containing 8M urea and 25 mM ammonium bicarbonate was added to the dry pellet, and the sample was resuspended by vortexing for 30 min at RT. 10 mM of tris (2-carboxyethyl) phosphine was added to the tubes and incubated at RT for 30 min 25 mM of iodoacetamide was added to the tubes and incubated at RT for 30 min in the dark. The proteins were then subjected to trypsin digestion (Trypsin:protein 1:20) at 37 °C overnight. The peptides were desalted using C-18, eluted in a fresh MCT, and stored at −80 °C.

### Protocol for Isolation of Membrane and Cytosolic Fractions

*Rv-vc*_*dc*_*, Rv-secA1*_*dc*_*,* and *Rv-tatA*_*dc*_ were grown in the presence and absence of ATc (500 ng/ml) in 7H9+OADC media in quadruplets. On the fourth day, the cells were pelleted and resuspended in lysis buffer (1× PBSG, 1× PMSF, and PIC: Protease Inhibitor Cocktail), and cell lysates were prepared using bead beating for eight rounds (1 min ON and 2 min OFF cycles on ice). Samples were centrifuged at 158,71*g* for 10 min at 4 °C. The supernatant was collected and subjected to ultracentrifugation at 1,000,00*g* for 2h at 4 °C. After the first round of ultracentrifugation, the supernatant was collected and designated as the Cytosolic fraction (C). The pellets were resuspended in 1× PBSG with 1× PMSF and subjected to another two rounds of ultracentrifugation to collect the membrane fractions (M). After the final (third) round of ultracentrifugation, the pellets (M) were resuspended in resuspension buffer (5% SDS, 0.1 M Tris-Cl pH-8.5) and stored at −80 °C (Supplemental Fig. S7*A*).

### MS Sample Preparation and Processing

25 μg of proteins from the membrane and cytosolic fractions were reduced and alkylated with 5 mM tris (2-carboxyethyl) phosphine and 50 mM iodoacetamide, respectively. Trypsin was added to the lysate at a 1:50 ratio (μg) and incubated at 37 °C for 16 h. The digested peptides were purified using a C-18 silica cartridge and dried under speed vacuum. The dry pellet was resuspended in buffer A (2% acetonitrile + 0.1% formic acid) and submitted to a mass spectrometer equipped with an Easy-nLC-1000 system connected to an Orbitrap Exploris. The C-18 column (15 cm/1.9 μm) was loaded with 1 μg of peptide and separated using a gradient of buffer B (80% acetonitrile + 0.1% formic acid) at a flow rate of 500 nl/min. The LC gradient duration was 110 min. The eluate was injected for MS analysis. MS1 spectra were acquired (Max IT = 60 ms, AGQ goal = 300%; RF Lens = 70%; R = 60K, mass range = 375 − 1500; profile data). All charge states for a given precursor were excluded for 30 s using dynamic exclusion. The top 20 peptides' MS2 spectra were acquired using the following parameters: AGC target 100%, R = 15K, and Max IT = 60 ms.

### Experimental Design and Statistical Rationale for Mass Spectrometry

For each strain four biological replicates (n = 4) were processed and 24 RAW files generated were annotated using Proteome Discoverer 2.5 against the *M. tuberculosis* (strain ATCC 25618/H37Rv) protein obtained from UniProt (proteome identifier UP000001584) database (39) comprising 2306 reviewed entries, downloaded on September 25, 2025. The three replicates (n = 3) were considered for further analysis. The precursor and fragment mass tolerances were set to 10 ppm and 0.02 Da for dual Sequest and Amanda searches. Trypsin/P was set as the protease specificity, with cleavage at the C terminus of "K/R " unless followed by "P". Carbamidomethyl on cysteine was set as a fixed modification, and oxidation of methionine and N-terminal acetylation were set as variable modifications for the database search. Peptides and proteins were annotated at High confidence, corresponding to a false discovery rate of 0.01. Label free quantification values were obtained from MaxQuant (40) as shown in *Gagan et al.* (41). Mycobrowser (42) database was used for creating a list of *Mtb* proteins with their respective UniProt ID, Locus ID, and functional classification, and was augmented with information on the presence or absence of SP, transmembrane helices, and non-classical secretion obtained by *in silico* prediction using SignalP v 6.0^34^, DeepTMHMM v 1.0 (https://services.healthtech.dtu.dk/services/DeepTMHMM-1.0/), Phobius v 10.1 (https://phobius.sbc.su.se/), and PncsHub (35). The UniProt IDs and the label free quantification values obtained from MS data were mapped with the list. Proteins with <2 unique peptides were removed, as this eliminates low-confidence identifications that may arise from random peptide matches. For CF samples, proteins with transmembrane helices or known vesicle-associated transport (based on PncsHub (35)) were excluded to avoid contamination from membrane fragments or vesicles, ensuring only genuinely secreted proteins were analyzed. For membrane fractions, proteins lacking transmembrane helices were removed, leaving only integral or strongly membrane-associated proteins. We compared both the CF and membrane fractions dataset obtained with previous studies to further enhance reliability by prioritizing consistently identified proteins, thereby reducing false positives and increasing confidence in the final dataset.

After filtration, the proteins and label free quantification values were log-normalized, and the differential abundance was performed using limma (43). Later, *p*-values were adjusted for multiple testing using the Benjamini–Hochberg method, and proteins with adjusted *p* < 0.05 were considered significant (Fig. 3*A*). The differential abundance, SP distribution, and functional annotation were visualized using volcano plots, alluvial plots, and chord plots, were generated using ggplot2 (https://ggplot2.tidyverse.org/), ggalluvial (44), and circlize (45) packages, respectively, in R.

**Fig. 2:**
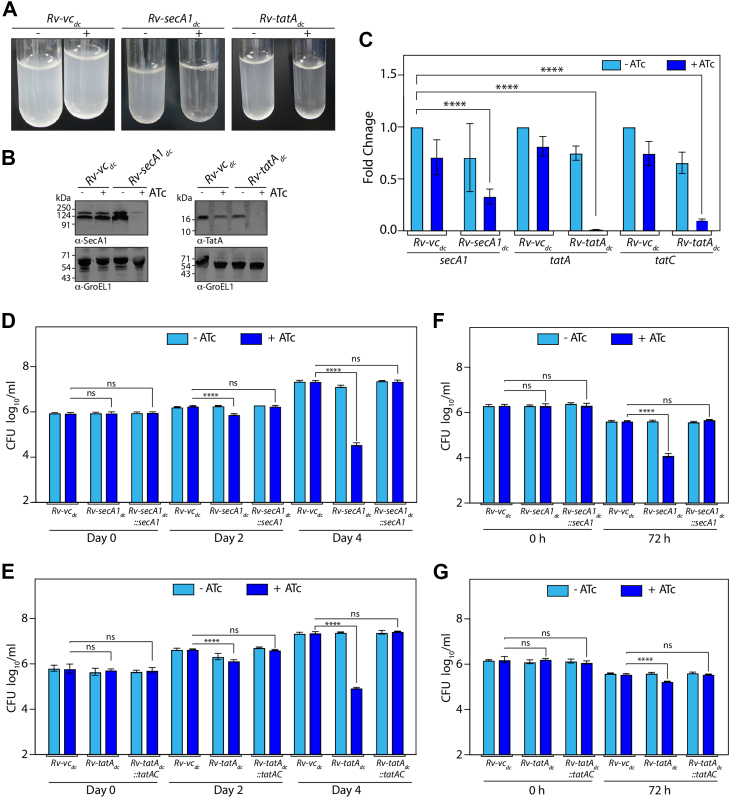
**Impact of bacterial survival upon *secA1* and *tatAC* depletion.***A*, *Rv-vc*_*dc*_, *Rv-secA1*_*dc*_*,* and *Rv-tatA*_*dc*_ were inoculated at A_600_ ∼0.1 and grown for 4 days in the presence and absence of ATc. The tubes represent the depletion of *Rv-secA1*_*dc*_ and *Rv-tatA*_*dc*_ as compared to *Rv-vc*_*dc*_. *B*, whole cell lysates were prepared from *Rv-vc*_*dc*_ + ATc, *Rv-secA1*_*dc*_ −/+ATc, and *Rv-tatA*_*dc*_ −/+ATc from a fourth day culture. 50 μg of each protein lysate was resolved on 10% (SecA1) and 15% (TatA) SDS-PAGE, transferred to a Nitrocellulose membrane, and probed with α-SecA1 (for SecA1 depletion) and α-TatA (for TatA depletion) antibodies generated in the lab. α-GroEL1 antibody was used as a loading control. *C,* total RNA was isolated from *Rv-vc*_*dc*_ −/+ATc, *Rv-secA1*_*dc*_ −/+ATc and *Rv-tatA*_*dc*_ −/+ATc from third day grown cultures for qRT-PCR. The expression of *secA1*, *tatA*, and *tatC* was normalized with respect to 16S rRNA, and the Fold Change (2^-ΔΔCt^) value (mean ± SD (n = 4)) was plotted using GraphPad Prism. The Statistical significance was analyzed using Two-way ANOVA ∗∗∗∗, *p* < 0.0001. *D* and *E*, the graph depicts the CFU of the *in vitro* survival of bacteria of *Rv-vc*_*dc*_−/+ATc, *Rv-secA1*_*dc*_−/+ATc, *Rv-secA1*_*dc*_*::secA1* −/+ATc, *Rv-tatA*_*dc*_−/+ATc and *Rv-tatA*_*dc*_*::tatAC* −/+ATc on day 0, day 2 and fourth day post depletion. GraphPad Prism software was used to plot CFUs and perform statistical analysis using Two-way ANOVA. Two independent experiments were performed as biological replicates, and each experiment was conducted in triplicate. Data represents mean and ±SD, ∗∗∗∗*p* < 0.0001. *F* and *G*, peritoneal macrophages, isolated from the mice peritoneum 4 days post thioglycolate injection, were infected with the *Rv-vc*_*dc*_*, Rv-secA1*_*dc*_*, Rv-secA1*_*dc*_*::secA1, Rv-tatA*_*dc*_*,* and *Rv-tatA*_*dc*_*::tatAC* at 1:5 MOI. 4 h post-infection, CFUs were enumerated at 0h and 72h. Survival of the *Rv-vc*_*dc*_*, Rv-secA1*_*dc*_*, Rv-secA1*_*dc*_*::secA1, Rv-tatA*_*dc*_*,* and *Rv-tatA*_*dc*_*::tatAC* in the present and absence of ATc were plotted using GraphPad Prism software and statistical analysis were performed using Two-way ANOVA. Two independent experiments were performed as biological replicates, and each experiment was conducted in triplicate. Data represents mean and ±SD, ∗∗∗∗*p* < 0.0001. CFU, colony forming unit.

**Fig. 3:**
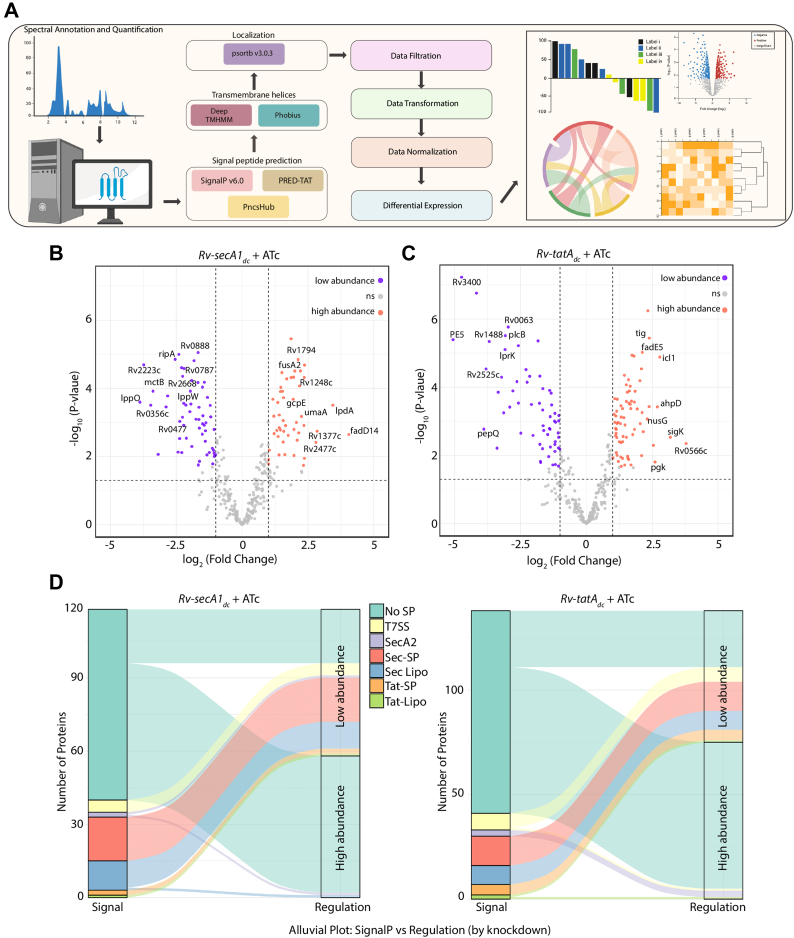
**Secretome of SecA1 and TatAC.***A*, schematic representation of proteome (Secretome and Membrane proteome) data analysis of SecA1 and TatAC. Raw data generated was searched against the UniProt database, and the resulting spectra were annotated for peptide and protein identification. Sec, Tat, and T7SS signal peptides in the proteins were identified using SignalP v6, PRED-TAT, and PncsHub, and transmembrane helices were predicted using DeepTMHMM 1.0 and Phobius. Protein localization was also checked using psortb v3.0.3. The proteins detected in the samples (n = 3 replicates for each strain) were filtered, normalized, differential abundance analysis and visulatization was done using R. Created in BioRender. Nandicoori, V. (2026) https://BioRender.com/i7bto44. *B* and *C,* the volcano plots of SecA1 (*B*) and TatAC (*C*) depletion with 1≥log2Fold Change ≤ −1. The *purple dots* depict the low-abundance proteins, the *orange dots* depict the high-abundance proteins, and the grey dots represent non-significant proteins. *D*, the Alluvial plots showing the number of proteins containing various signal peptides and their differential abundances upon SecA1 and TatAC depletion.

### Scanning Electron Microscopy

For Scanning Electron Microscopy, 5 ml cells at A_600_ ∼0.5 of *Rv-vc*_*dc*_*, Rv-secA1*_*dc*_*, Rv-secA1*_*dc*_*:: secA1, Rv-tatA*_*dc*_*,* and *Rv-tatA*_*dc*_*::tatAC* were centrifuged at 3500×*g* for 10 min at RT. The supernatant was discarded, and the pellets were resuspended in fixative. The cells were incubated at 37 °C. The cells were centrifuged for 10 min, and the pellets were washed twice with 0.1 M Na-Cacodylate buffer. The cells were resuspended in 1% OsO_4_ (Osmium tetroxide) and incubated at RT for 90 min in a twirler. The cells were centrifuged again for 10 min at RT. The supernatant was discarded, and dehydration was performed gradually with different concentrations of ethanol: 25% ethanol for 5 min, 50% ethanol for 7 min, 75% ethanol for 10 min, 95% ethanol for 20 min, and 100% ethanol for 30 min, three times. After each step, samples were centrifuged for 10 min at RT. The pellets were resuspended in Hexamethyldisilazane. The samples were gold-coated and imaged by Scanning Electron Microscopy (S3400 N, Hitachi). Cell lengths were measured using ImageJ, and the data were plotted in GraphPad Prism.

### Transmission Electron Microscopy (TEM)

For transmission electron microscopy (TEM) sample preparation, the Scanning Electron Microscopy protocol mentioned above was followed until the dehydration step. After that, the pellets were resuspended in propylene oxide and incubated at 4 °C for 1 to 2 h on a rotator. The samples were spun down, and the supernatant was discarded. A mixture of propylene oxide and resin in a 1:1 ratio was added to the samples, which were then incubated at 4 °C for 2 h on a rotator. This step was repeated with propylene oxide-to-resin ratios of 1:2 and 1:3. Finally, pure resin was added to the samples, which were incubated overnight at 4 °C on a rotator. The next day, the resin was discarded, and pure resin was added to the samples, which were incubated at 60 °C for 2 h. The samples were then sectioned on a microtome into 70 nm-thick transverse sections. The sections were stained with uranyl acetate, and images were acquired using TEM (Talos L120c, Thermo Fisher Scientific).

### Ethidium Bromide (EtBr) Uptake Assay

*Rv-vc*_*dc*_*, Rv-secA1*_*dc*_*, Rv-secA1*_*dc*_*::secA1, Rv-tatA*_*dc*_*,* and *Rv-tatA*_*dc*_*::tatAC* strains were inoculated with and without ATc, and *Rv-pptr-B* was grown in the presence and absence of 250 ng/ml Pristinamycin at A_600_ ∼0.1. A heat-killed *Rv-vc*_*dc*_ was used as a purposefully dead cell control. The heat-killed cells were prepared by heating *Rv-vc*_*dc*_ at 80 °C for 90 min in a water bath. The cells were then harvested at five time points, D0 (day 0), D2, D3, D4, and D5, for the EtBr uptake assay. The cell pellets were washed with 1× PBST_80_. Cells at A_600_ ∼0.5 were harvested from the cultures in an MCT, and 1 μg/ml EtBr was added with and without Verapamil (40 μg/ml). The cultures were incubated at 37 °C for 15 min (data not shown) and 30 min in a standing incubator in the dark. After incubation, the cells were washed twice with 1× PBST_80_ to remove residual EtBr. After the second wash, the pellets were resuspended in 4% paraformaldehyde for fixation. 200 μl of the cell suspensions were transferred into a 96-well plate for fluorescence readings. The cell suspensions were excited at 510 nm, and the emission spectra were collected at 595 nm. Without EtBr, the added cells were taken as a control to normalize and obtain the absolute unit value using GraphPad Prism software (https://www.graphpad.com/features).

### Propidium Iodide (PI) Uptake Assay

*Rv-vc*_*dc*_*, Rv-secA1*_*dc*_*, Rv-secA1*_*dc*_*::secA1, Rv-tatA*_*dc*_*, and Rv-tatA*_*dc*_*::tatAC* strains were grown in the presence and absence of ATc, and *Rv-pptr-B* was grown in the presence and absence of 250 ng/ml Pristinamycin. The cells were processed at time points D0, D2, D3, D4, and D5 for a propidium iodide (PI) uptake assay. A heat-killed *Rv-vc*_*dc*_ strain was used as a purposefully dead cell control (as mentioned above). The cells were washed with 1× PBST_80_. A volume of 5 μg/ml PI was added to cells at A_600_ ∼0.5, with and without Verapamil (40 μg/ml), and the cells were incubated at 37 °C for 5 min in a standing incubator in the dark. After incubation, the cells were washed twice with 1× PBST_80_ to remove any residual PI. The cells were then resuspended in 4% paraformaldehyde. The cell suspension was used for flow cytometry analysis. The fluorescence of the PI-containing cells was plotted using GraphPad Prism.

## Results

### Computational Reconstruction of Protein Secretion Systems in *Mtb*

The computational reconstruction of the secretory systems in *Mtb* was inspired by *Feizi et al.* (32), who developed a genome-scale reconstruction of the protein secretion system in the yeast *S. cerevisiae* to elucidate context-specific regulation and pathway activity, offering insights into how the secretion system behaves under various physiological conditions. We employed a similar reconstruction approach to integrate molecular components and mechanistic interactions, thereby developing a visual framework that encapsulates the secretion process at the systems level. A total of 92 key secretory components belonging to three subsystems: Sec-dependent (SRP-mediated, SecA2-and SecA1-mediated), Sec-independent (Tat), and T7SS (Supplemental Table S1*A*), were identified (Supplemental Fig. S1*A*). Among these, 19 components were unique to Sec pathways, three to Tat, and 67 to T7SS, respectively (Supplemental Fig. S1*B*). Four components: LepB (Rv2903c), Lgt (Rv1614), LspA (Rv1539), and Ppm1 (Rv2051c), are shared between the Sec and Tat pathways and are primarily involved in lipidation and SP cleavage. Five components, primarily chaperones (Rv3417c- GroEL1, Rv0440- GroEL2, Rv3418c- GroES, Rv0009- PpiA, and Rv2582- PpiB), were common to both the Tat and T7SS pathways (Supplemental Fig. S1*b*).

A total of 198 mechanistic reactions that illustrate the step-by-step procedure involved in protein export were utilized to generate a predictive computational model of secretion systems in *Mtb* (Fig. 1, Supplemental Figs. S2, and S3). Protein export *via* SRP involves recognizing the N-terminal SRP SP, translocating the protein through a membrane channel, and cleaving the SP to yield a mature protein (Supplemental Fig. S3). In the absence of the SRP signal, alternative post-translational routes, such as the SecA1-dependent pathway or the Tat system, are used (Supplemental Fig. S2 and Supplemental Table S2*A*). SecA2 is predominantly cytosolic and exports proteins in conjunction with the chaperone SatS (46). SecA2 reactions were not included in the model, as there is no clear evidence for how these proteins are transported. In addition, *Mtb* also contains ESX-1 to ESX-5 (47) secretion systems that facilitate the transport of virulent proteins across the membrane (48, 49), and studies have demonstrated that components of the ESX systems create a channel in the inner membrane (Fig. 1*C* and Supplemental Table S2), although the mechanism by which the proteins are exported into the extracellular environment remains unclear.

Integrative analysis of publicly available ChIP-Seq data (36, 50), together with the secretory components identified in this study, revealed 68 TFs from 28 families that directly regulate secretion-associated genes. Notably, Rv0081 (ArsR family) regulated 12 components across the Sec, Tat, and T7SS pathways, while CosR (Rv0967) and TrcR (Rv1033c) regulated 11 and nine components, respectively (Supplemental Fig. S4, *A* and *B*). Among TFs regulating two to four components, members of the TetR and WhiB families predominated, mainly influencing T7SS components (Supplemental Fig. S4*C*). To assess transcriptional responses under stress, RNA sequencing data (37) were integrated with the secretion components. Starvation and SDS stress caused the most pronounced changes, with Sec and Tat components largely downregulated during starvation, whereas T7SS components were upregulated (Supplemental Fig. S4*D*).

### Compromised Bacterial Survival upon *secA1* and *tatAC* Depletion

**Figure S5:**
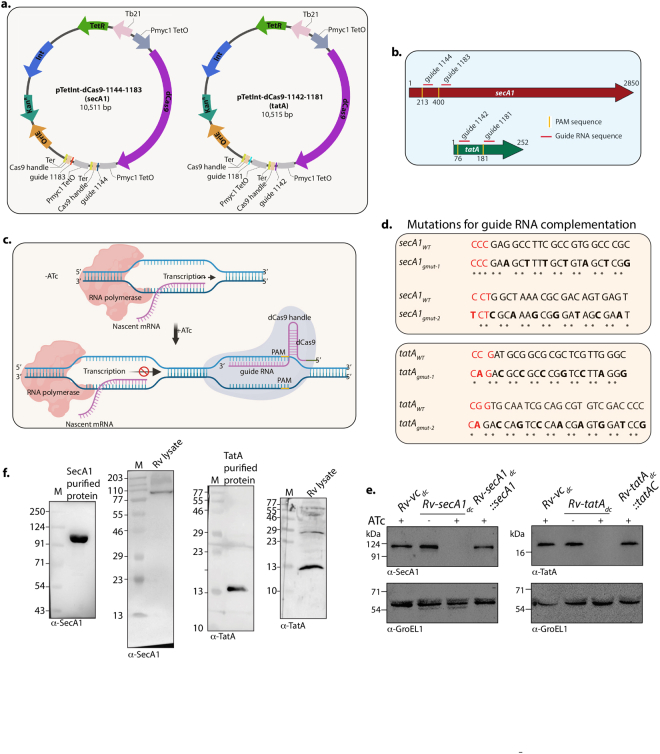


T7SS systems in *Mtb* are extensively investigated in relation to other secretion pathways, specifically the Sec and Tat pathways. Therefore, in this report, we aim to focus on and investigate the SecA1 and Tat secretion pathways; thus, we selected SecA1 and TatA as the candidates. High-throughput transposon mutagenesis studies indicated that both *secA1* and *tatA* are essential for *in vitro* growth in complete media (51); thus, we generated CRISPRi-mediated knockdown strains (30) (*Rv-secA1*_*dc*_ and *Rv-tatA*_*dc*_) in *H37Rv*. Depletion of SecA1 and TatA upon ATc addition (day 4) resulted in impaired growth *in vitro, as* confirmed by Western blotting with α-SecA1 and α-TatA antibodies (Fig. 2, *A* and *B*). The CRISPRi system is known to cause polarity effects for the entire operon (30). *SecA1* is a solo gene, while *tatA* and *tatC* form a two-gene operon, and both are essential *in vitro*. Although we were targeting only *tatA*, the addition of ATc led to decreased levels of both *tatA* and *tatC* (Fig. 2*C*). Thus, we used *tatA*_*gmut*_ alongside *tatC* to complement *Rv-tatA*_*dc*_ and generated complementation strains (*Rv-tatA*_*dc*_*::tatAC*) (Supplemental Fig. S5*E*). In the *Rv-secA1*_*dc*_ + ATc strain, compromised growth was observed around ∼4 log_10_ fold as compared to *Rv-vc*_*dc*_ + ATc after 4 days of depletion *in vitro* (Fig. 2*D*). While in the case of *Rv-tatA*_*dc*_ + ATc, the growth defect was noticed around ∼3 log_10_ fold (Fig. 2*E*). In the peritoneal macrophages, the growth was compromised up to ∼2.5 log_10_ fold in *Rv-secA1*_*dc*_ + ATc and ∼0.8 log_10_ fold in *Rv-tatA*_*dc*_ + ATc as compared to *Rv-vc*_*dc*_ + ATc control (Fig. 2, *F* and *G*). The functional complementation rescued the growth phenotype in both *in vitro* and *ex vivo*. We conclude that we successfully generated the *Rv-secA1*_*dc*_ and *Rv-tatA*_*dc*_ knockdown strains, indicating that *secA1* and *tatAC* (*tatA* and *tatC*) are essential for bacterial survival.

### Change in the Secretome upon SecA1 and TatAC Depletion

**Figure S6:**
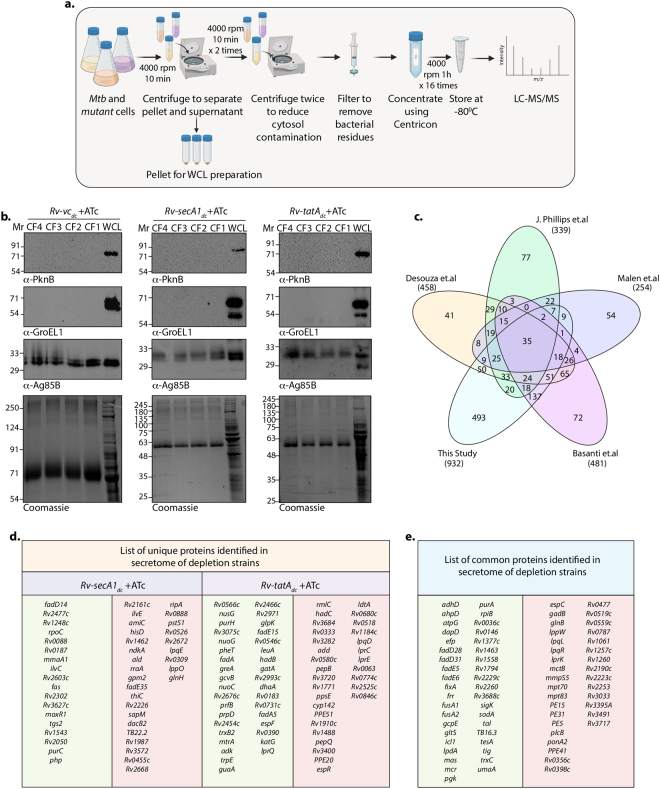


*In silico* studies predict putative Sec or Tat substrates based on the presence of consensus SPs or twin-arginine motifs, respectively; no experimental studies confirm these predictions. To identify the SecA1-and Tat pathway mediated secretome, the supernatant of *Rv-vc*_*dc*_, *Rv-secA1*_*dc*_*,* and *Rv-tatA*_*dc*_ strains was enriched for the CF proteins (38) and the pellet was processed for WCL (Supplemental Fig. S6*A*). When both the fractions were probed with α-PknB and α-Ag85 B antibodies (markers for membrane/WCL and secreted proteins), the absence of a band corresponding to PknB and the presence of Ag85 B bands in CF fractions (Supplemental Fig. S6*B*) are indicative of the lack of contamination from other fractions. Ag85 B is shown to be secreted through the SecA pathway (52). As anticipated, we observed lower Ag85 B levels upon SecA1 depletion; however, we also observed decreased Ag85 B levels upon TatAC depletion (Supplemental Fig. S6*B*).

As outlined in Figure 3*A*, processing the CF data yielded 1255 proteins present in all three samples; after applying the filters described in the methods, 932 proteins were retained. We hypothesized that the absence of SecA1 or TatAC would result in a substantial decrease in the number of secreted proteins compared to the *Rv* control. On the contrary, we observed a higher number of secreted proteins, including known cytosolic proteins, in both samples, suggesting changes in overall cellular homeostasis upon depletion (Supplemental Table S5). To minimize false positives and enhance confidence, 410 secretory proteins that were common between the current study and at least one other previously published study (four previous studies (53, 54, 55, 56)) were retained (Supplemental Fig. S6*C*). We obtained a total of 134 differentially secreted proteins in *Rv-secA1*_*dc*_ + ATc (Fig. 3*B*) and 158 proteins for the *Rv-tatA*_*dc*_ + ATc samples (Fig. 3*C*). Of these, 118 (58 higher and 60 lower abundances) and 138 (75 higher and 63 lower abundances) showed a *p*-value <0.05 in *Rv-secA1*_*dc*_ + ATc and *Rv-tatA*_*dc*_ + ATc samples, respectively (Supplemental Table S5) with 72 proteins common to both (Supplemental Fig. S6*E*). Proteins lowered only in *Rv-secA1*_*dc*_ + ATc (27) or *Rv-tatA*_*dc*_ + ATc (30) were considered to be probable SecA1 or Tat substrates, respectively, while 33 were shared, suggesting dual dependence or indirect effects (Supplemental Fig. S6*D*) such as changes in the cellular homeostasis. Among the 27 probable SecA1 specific proteins, 13 had SecA1 SP and SecA1 SP + LM (Fig. 3*F*). Of the 30 probable Tat pathway specific proteins, only five had Tat SP and Tat SP + LM (Fig. 3*D*). We speculate that depletion of the SecA1 and TatAC, altered the export of membrane stabilizing proteins, potentially compromising *Mtb* membrane integrity and leading to leakage of periplasmic or cytoplasmic proteins into the CF fraction. To address this, we investigated the changes in the membrane proteome upon SecA1 and TatAC depletion.

### Mycobacterial Membrane Proteome Alteration owing to SecA1 and TatAC Depletion

**Figure S7:**
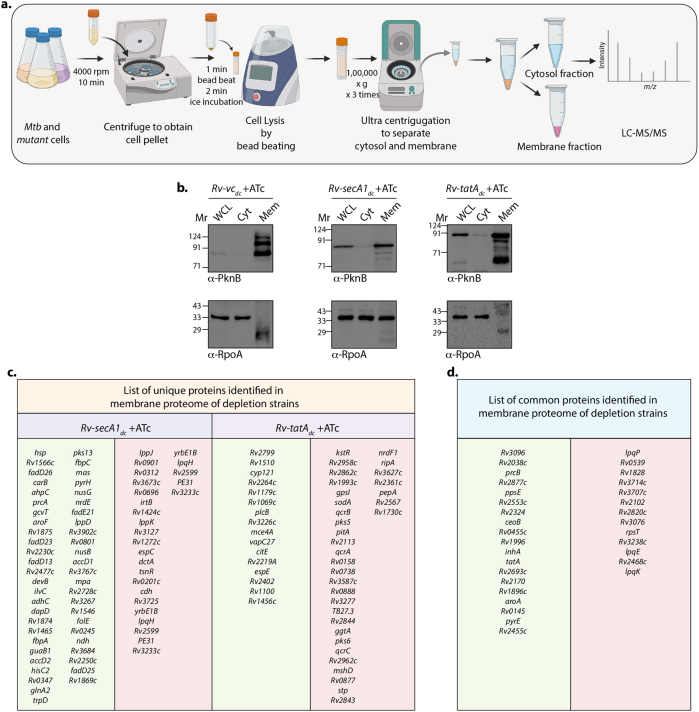


The cytosolic and membrane fractions from *Rv-vc*_*dc*_ + ATc, *Rv-secA1*_*dc*_ + ATc, and *Rv-tatA*_*dc*_ + ATc samples were probed with α-RpoA (a cytosolic marker) and α-PknB (a membrane marker) antibodies, respectively. As anticipated, PknB was detected in WCL and the membrane fraction but not in the cytosolic fraction, whereas RpoA was detected in WCL and the cytosolic fraction but not in the membrane fraction in *Rv-vc*_*dc*_ + ATc samples. This suggested that the fractionation method was appropriate and yielded enrichment. However, in the fractions prepared from *the Rv-secA1*_*dc*_ + ATc sample, RpoA was also detected in the membrane fraction. This was not the case in fractions prepared from *Rv-tatA*_*dc*_ + ATc or in the control *Rv-vc*_*dc*_ + ATc samples (Supplemental Fig. S7*B*).

**Fig. 4:**
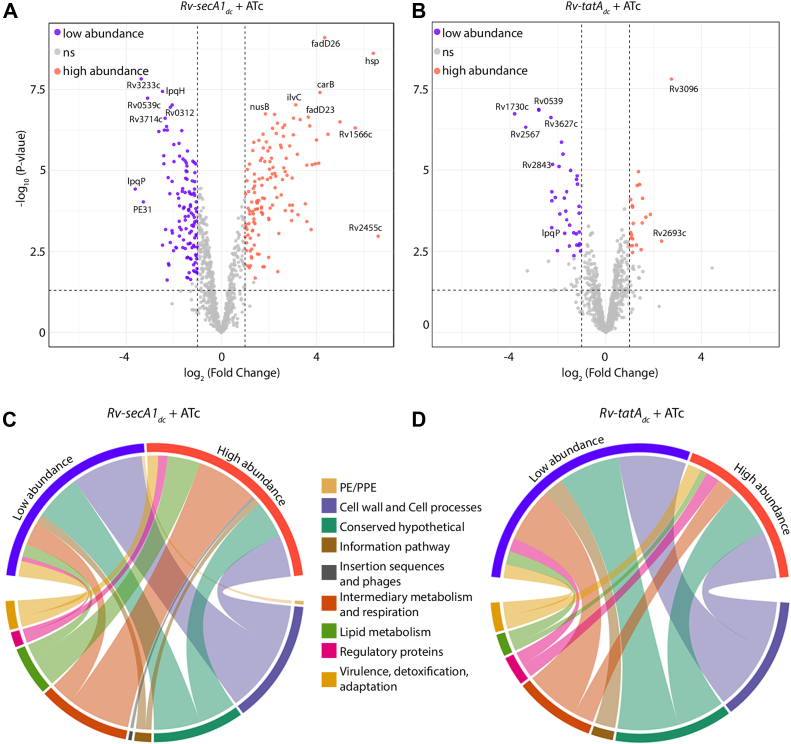
**Membrane proteome of SecA1 and TatAC-depleted cells.***A* and *B*, the volcano plots representing the proteins detected in the samples (n = 3 replicates for each strain) affected by SecA1 and TatAC depletion using padj<0.05 and 1≥log2Fold Change ≤ −1. The *purple dots* depict low-abundance proteins, the *orange dots* high-abundance proteins, and the *gray dots* non-significant proteins. The chord plot depicts the differential abundances of high and low proteins upon the (*C*) SecA1-depleted condition and (*D*) TatAC-depleted condition grouped by functional categories.

A total of 2335 proteins were detected in the membrane fractions *of Rv-vc*_*dc*_ + ATc, *Rv-secA1*_*dc*_ + ATc, and *Rv-tatA*_*dc*_ + ATc samples. To enhance confidence, we retained only those candidates that were common to both the current study and at least one other previously published study (filtered as shown in methods (22, 57), reducing the number to 943 proteins. A total of 260 proteins were differentially abundant in *Rv-secA1*_*dc*_ + ATc (138 higher and 122 lower abundances) (Fig. 4*A*), and the number in *Rv-tatA*_*dc*_ + ATc was much lower, accounting for 81 in total (31 high and 50 low abundance) (Fig. 4*B*). In addition, more cytosolic proteins were detected in the membrane fraction of *Rv-secA1*_*dc*_ + ATc than in *Rv-tatA*_*dc*_ + ATc. This was also reflected in the Western blot (Supplemental Fig. S7*B*), where cytosolic markers were detected in *Rv-secA1*_*dc*_ + ATc samples, but not in *Rv-tatA*_*dc*_ + ATc. A total of 32 differentially abundant proteins were common in both the samples; among these, Phenolpthiocerol synthesis type-I polyketide synthase, Proteasome beta subunit, Rv0455c, and Rv1896c were highly abundant in *Rv-secA1*_*dc*_ + ATc, whereas they were low in *Rv-tatA*_*dc*_ + ATc (Supplemental Table S6). Functional annotation of differentially abundant membrane fraction proteins showed that proteins belonging to cell wall and cell processes were low in abundance, while those involved in metabolic processes were high abundant in *Rv-secA1*_*dc*_ + ATc (Fig. 4*C*). However, both cell wall and cell process proteins, along with metabolic process proteins, were low in abundance in *Rv-tatA*_*dc*_ + ATc (Fig. 4*D*). Together, the data suggests that there may be a possible membrane dysbiosis in *Rv-secA1*_*dc*_ + ATc samples, which may also be the case with *Rv-tatA*_*dc*_ + ATc, but at a lower scale.

### SecA1 and TatAC Depletion Result in Mycobacterial Membrane Permeability

**Fig. 5:**
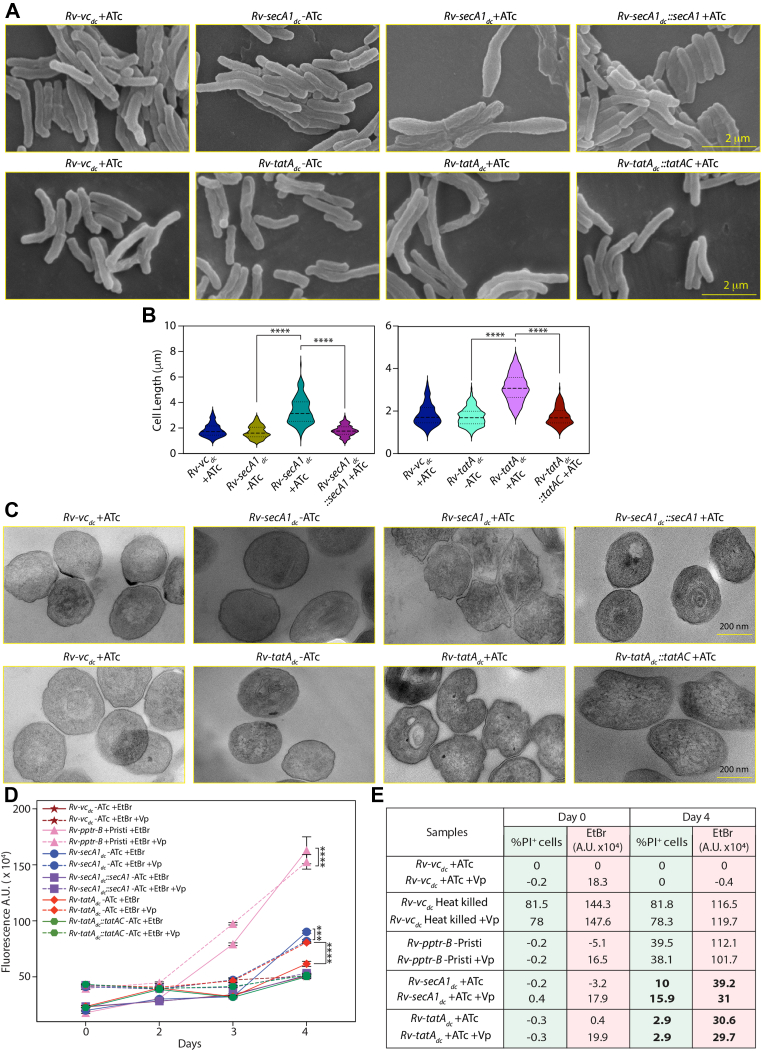
**Alteration of the cellular morphology upon SecA1 and TatA depletion.***A*, scanning Electron Microscopy images of *Rv-vc*_*dc*_ + ATc*, Rv-secA1*_*dc*_ −/+ATc*, Rv-secA1*_*dc*_*::secA1* +ATc*, Rv-tatA*_*dc*_ −/+ATc, and *Rv-tatA*_*dc*_*::tatAC* + ATc. *Rv-vc*_*dc*_ + ATc*, Rv-secA1*_*dc*_ −/+ATc*, Rv-secA1*_*dc*_*::secA1* +ATc*, Rv-tatA*_*dc*_ −/+ATc, and *Rv-tatA*_*dc*_*::tatAC* + ATc were grown for 4 days post ATc addition, and cells were fixed and processed for Scanning Electron Microscopy.. Images of cell morphology were obtained by Scanning Electron Microscopy at 100,00× magnification. The scale bar represents 2 μm. *B*, cell length (μm) for both SecA1 and TatAC was measured, quantified using ImageJ software (https://imagej.net/ij/)and plotted using GraphPad Prism software. The statistical analysis was performed by One way ANOVA, ∗∗∗∗*p* < 0.0001. *C*, transmission electron microscopy images of *Rv-vc*_*dc*_ + ATc*, Rv-secA1*_*dc*_ −/+ATc*, Rv-secA1*_*dc*_*::secA1* +ATc*, Rv-tatA*_*dc*_ −/+ATc, and *Rv-tatA*_*dc*_*::tatAC* + ATc. *Rv-vc*_*dc*_ + ATc*, Rv-secA1*_*dc*_ −/+ATc*, Rv-secA1*_*dc*_*::secA1* +ATc*, Rv-tatA*_*dc*_ −/+ATc, and *Rv-tatA*_*dc*_*::tatAC* + ATc were grown for 4 days post ATc addition, and cells were fixed and processed for transmission electron microscopy. The images of the cell membrane were taken at 450,00× magnification. Scale bar 200 nm. *D*, the graph represents the fluorescence Arbitrary Unit (A.U. x10^4^) of EtBr uptake by the control *Rv-vc*_*dc*_ + ATc, and the depleted cells *i.e. Rv-pptr-B* -pristi, *Rv-secA1*_*dc*_ + ATc, *Rv-secA1*_*dc*_*::secA1* +ATc, *Rv-tatA*_*dc*_ + ATc, *Rv-tatA*_*dc*_*::tatA* + ATc strains at time points D0, D2, D3, and D4. The filled lines depict only EtBr uptake by the strains and the dotted lines depict the EtBr uptake in the presence of Verapamil. GraphPad Prism software was used to plot A.U.s at different time points and perform statistical analysis using Two-way ANOVA. Two independent experiments were performed as biological replicates, and each experiment was conducted in triplicate. Data represents mean and ±SD, ∗∗∗∗*p* < 0.0001. *E*, the table depicts the % of PI^+^ cells and EtBr uptake (A.U. x10^4^) by the *Rv-vc*_*dc*_ + ATc, heat-killed *Rv-vc*_*dc*_*, Rv-pptr-B* -pristinamycin, *Rv-secA1*_*dc*_ + ATc and *Rv-tatA*_*dc*_ + ATc on day 0 and 4. Vp stands for addition of Verapamil (40 μg/ml). The values shown in the table was after deducting the background uptake observed with *Rv-vc*_*dc*_ + ATc. Two independent biological experiments were performed, and the average values from the two independent experiments are shown in the table. EtBr, ethidium bromide.

Figures 3 and 4 show significant alterations in the secretome and membrane proteome following SecA1 and TatAC depletion. Notably, several cytosolic proteins appeared in both fractions, and many proteins were shared between the two pathways. These observations suggest that SecA1 and, to a lesser extent, TatAC depletion may compromise membrane integrity. Scanning electron microscopy performed to investigate the phenotypic impact on cellular morphology showed bulged and elongated cells (>40% cells were >3 μm) upon SecA1 depletion, which could be restored by complementation (Figs. 5, *A* and *B* and Supplemental Fig. S8, *A* and *C*). While the cells were elongated (>10% cells were >4 μm) compared with *Rv* upon TatAC depletion, and did not show a bulged phenotype like *Rv-secA1*_*dc*_ + ATc (Fig. 5, *A* and *B* and Supplemental Fig. S8*B*). To further investigate the effect of SecA1 and TatAC depletion on the ultrastructure of the *Mtb* membrane, we performed TEM. We observed that the architecture of the cell membrane was significantly altered upon SecA1 or TatAC depletion (Fig. 5*C*). However, we did not detect any membrane perforations, suggesting that the possible changes at this time point may be more subtle and possibly more permeable.

To investigate changes in membrane permeability, we used heat-killed *Rv-vc*_*dc*_ (HK*-Rv-vc*_*dc*_) cells as purposefully dead cells. We also used *Rv-pptr-B* (essential kinase PknB can be depleted upon withdrawing the inducer pristinamycin). PknB is known to regulate cell division and cell wall synthesis and hence can also serve as a control for cell death. We measured the EtBr fluorescence and plotted the relative values at different time points for *Rv-vc*_*dc*_ + ATc, heat-killed *Rv-vc*_*dc*_ (HK*-Rv-vc*_*dc*_), *Rv-pptr-B* -pristi (PknB Knockdown), *Rv-secA1*_*dc*_ + ATc and *Rv-tatA*_*dc*_ + ATc in the presence and absence of Verapamil (40 μg/ml) (Fig. 5*D*). We observed significant cell death with both SecA1 and TatAC depletion on day 5, and hence we limited our analysis to the first 4 days (day 0, day 2, day 3 and day 4).

On day 0, no death was observed in any strain, and accordingly, EtBr uptake was negligible (after deducting the background uptake observed with *Rv-vc*_*dc*_ (Fig. 5, *D* and *E* and Supplemental Fig. S8, *D* and *E*). The addition of Verapamil increased EtBr fluorescence, clearly demonstrating that blocking efflux pumps increased accumulation in all cases. On day 4, uptake per se was higher, even in the control *Rv-vc*_*dc*_ strain (Supplemental Fig. S8, *D* and *E*), and did not increase further upon Verapamil addition. This suggests that the mycobacterial membrane has become more permeable on day 4 compared with day 0, and that the impact of blocking efflux pumps is no longer apparent. HK*-Rv-vc*_*dc*_ showed significant EtBr uptake, suggesting compromised membrane integrity due to cell death (80% death). Even though cell death observed upon PknB depletion (*Rv-pptr-B* -pristinamycin) is around 40%, it also showed EtBr uptake comparable to HK-*Rv-vc*_*dc*_ (heat-killed *Rv-vc*_*dc*_). This may be due to both compromised membrane homeostasis and cell death. In the SecA1-depleted condition, cell death was 10 to 15%; however, EtBr uptake was ∼33% compared with HK-*Rv-vc*_*dc*_, suggesting that the accumulation is due to a combination of changes in membrane permeability and cell death. Importantly, in the case of TatAC depletion, we observed EtBr uptake of ∼33% compared with HK-*Rv-vc*_*dc*_, even though the observed cell death was only 2 to 3%, indicating that the observed uptake is mostly due to increased permeability.

## Discussion

The first genome-scale model for protein secretion was developed for yeast, *S. cerevisiae,* using a bottom-up approach with the Protein Specific Information Matrix framework, providing a systems-level understanding of secretion machinery in eukaryotes (32). Subsequent studies have performed a genome-scale analysis of protein secretion systems in *Aspergillus oryzae* (58), humans (59, 60), mice (iMM1685s), and CHO cells (iCHO2048s) (60). In contrast, most available genome-scale models for prokaryotes primarily focus on metabolic pathways and lack detailed representations of protein secretion machineries and their regulation (61, 62, 63). The available metabolic models of *Mtb* (iNJ661 (64), GSMN-TB (65), iEK1011 (66), and sMtb2018 (67)) have not been extended to build an integrative model with a detailed mechanistic description of the protein secretion machinery at the systems level. While reports offer detailed mechanistic insights, the modular organization of T7SS (68) and the characterization of SecA2 (21) are available independently; however, a single, integrated reconstruction of *Mtb* secretion pathways (Sec, Tat, and ESX) has not been established. Here, we provided a pictorial and conceptual reconstruction of secretion systems in *Mtb*, encompassing 92 secretory components and 198 mechanistic reactions, to visualize the elements of the secretory machinery and their architecture within a systems-level model (Fig. 1 and Supplemental Figs. S1–S3). By integrating transcriptomic and ChIP-Seq datasets, we identified key transcription factors that regulate the expression of secretion system genes (Supplemental Fig. S4), revealing the dynamic regulatory networks that underpin their function, which was absent in most previous studies (68, 69, 70). In the future, our computational reconstruction of *Mtb* secretion systems can be expanded to create a proteome-constrained, genome-scale protein secretory model for *Mtb*, similar to the recently developed yeast model (71).

CRISPRi-mediated knockdown has limitations for operonic gene targets. While *secA1* is a single-gene operon, the *tatC* gene is downstream of *tatA*. Our qRT-PCR experiments showed that the expression of both *tatA* and *tatC* was downregulated upon induction of guide RNA and dCas9 (Fig. 2*C*). Since both TatA and TatC function in the same secretion pathway, we did not attempt to create a specific TatA depletion strain by complementing with *tatC* at another integrative locus. The number of secretory proteins identified in several major studies ranged from 257 (53), 254 (54), 481 (56) to 932 in the current study, and the increased numbers may be due to technical improvements in the mass spectrometer’s sensitivity (Fig. 3). A prior study in *M. marinum* identified 30 proteins, including 24 PE/PPE proteins that depended on the presence of ESX-5 (72). Another study identified 66 proteins that were either more or less abundant in the *ΔsecA2* mutant compared with the WT (22). Here, we report, for the first time, that secretion of 118 proteins was dependent on SecA1 and 138 on TatAC systems, respectively (Fig. 3). Interestingly, 72 proteins were commonly affected in both depletion strains (Supplemental Fig. S6), suggesting either shared pathway dependence or effects related to membrane perturbation. Upon SecA1 depletion, lipoproteins, which are typically localized to the membrane and contain classical Sec SPs, were found to be reduced in abundance (Fig. 3). Proteins like RipA, a PG endopeptidase (73), and Ami1, an amidase (74) necessary for maintaining PG synthesis, were also lower in abundance (Fig. 3). In *Streptomyces coelicolor*, the authors identified 43 Tat pathway substrates, 25 of which were validated using a sensitive agarase reporter assay (75). *Mtb* utilizes the Tat pathway to export various proteins, including enzymes, hydrolases, and proteins involved in cell wall processes (17). Tat-deficient mutants in *Msm* failed to export β-lactamases such as BlaS to the cell envelope, resulting in sensitivity to β-lactam antibiotics (76). Rv2525c also plays a role in maintaining cell wall integrity and stress adaptation in *Mtb* (77). We observed lower abundances of Rv2525c and PlcB proteins in the Tat-depleted secretome.

A total of 528 (78), 1417 (79), 1318 (22), and 1539 (57) membrane proteins were previously obtained from membrane proteome studies conducted by various groups, and 2335 were identified in our current study. This variation in the number may be due to the type of membrane fractionation methods and biological conditions used. Among 943 membrane proteins identified, 260 were differentially abundant in SecA1, and 81 were for TatAC depletion (Fig. 4). A previous study showed that 13 out of 15 solute binding proteins (SBPs), 12 out of 21 Mce transporter family members were reduced, and 13 out of 21 identified DosR-regulated proteins were increased in the *ΔsecA2* mutant (22). Here, we observed that upon SecA1 depletion, metabolic proteins, mostly those involved in lipid degradation, were highly abundant, while cell wall synthesis proteins were less abundant (Fig. 4). A similar trend was observed in TatAC-depleted cells. A previous study showed the localization of LpqH, a membrane-anchored lipoprotein (80), which was also found to be lower in abundance upon SecA1 depletion in the membrane fraction, thus validating the result (Supplemental Fig. S7, *C* and *D*).

A notable outcome of this study is the detection of numerous cytosolic proteins in the secretome (Supplemental Fig. S6, *D* and *E*) and membrane proteome (Supplemental Fig. S7, *C* and *D*) following SecA1 or TatAC depletion. These proteins were not analyzed further because they were not part of the earlier studies. Our ultrastructural analyzes TEM and the EtBr and PI uptake data (Fig. 5 and Supplemental Fig. S8) indicate that increased uptake of EtBr is likely due to both cell death and increased permeability in the case of SecA1 depletion and largely due to compromised permeability in the case of TatAC depletion, leading to the unintended release of periplasmic and cytoplasmic proteins. In line with these observations, in the membrane proteomics data, we have observed that most of the transporters, including phosphate uptake (PstS1, PstS2, PstS3), nutrient ABC transporters (ModC, CysA1), and dicarboxylate transporters (DctA), are reduced in abundance in the membrane fraction of the SecA1-depleted cells (low abundance but not significant in TatAC). However, we have not observed reduced abundance of efflux pump proteins in the membrane fractions of both the depleted cells. While Sec and Tat pathways are mechanistically distinct, some proteins can utilize either pathway, reflecting SP flexibility (6) and crosstalk between pathways. These findings underscore the importance of coordination and regulatory interplay among secretion systems, which ensures balanced export of proteins critical for mycobacterial virulence, adaptation, and physiology.

## Data Availability

The mass spectrometry data generated in this study have been deposited in PRIDE under accession PXD066777.

Any additional information required to reanalyze the data reported in this paper is available from the lead contact (vinaykn@nii.ac.in or vinaykn.ccmb@csir.res.in) upon request.

## Supplemental data

This article contains supplemental data (81, 82, 83, 84, 85, 86, 87, 88, 89, 90, 91, 92, 93, 94, 95, 96, 97, 98, 99, 100, 101, 102, 103, 104, 105, 106, 107, 108, 109, 110, 111, 112, 113, 114, 115, 116, 117, 118, 119, 120, 121, 122, 123, 124, 125, 126, 127, 128, 129, 130, 131, 132, 133, 134, 135, 136, 137, 138, 139, 140, 141, 142, 143, 144, 145, 146, 147, 148, 149, 150, 151, 152, 153, 154, 155, 156, 157, 158, 159, 160, 161, 162, 163, 164, 165, 166, 167, 168, 169, 170, 171, 172, 173, 174, 175, 176, 177, 178, 179, 180, 181, 182, 183, 184, 185, 186, 187, 188, 189, 190, 191, 192, 193, 194, 195, 196, 197).

## Conflict of interest

The authors declare no competing interests.

## Figures and Tables

**Figure S8:**